# Comparison of the efficacy and safety of rivaroxaban and low-molecular-weight heparin in Chinese lung cancer patients with nonhigh-risk pulmonary embolism

**DOI:** 10.1186/s12959-023-00453-y

**Published:** 2023-02-02

**Authors:** Yijun Song, Dawei Yang, Dongni Hou, Jun She, Yuanlin Song

**Affiliations:** 1 Department of Pulmonary and Critical Care Medicine, Zhongshan Hospital, Fudan University, 180 Fenglin Road, Shanghai, 200032 China; 2 Department of Pulmonary and Critical Care Medicine, Zhongshan Hospital (Xiamen), Fudan University, Xiamen, China; 3 Shanghai Engineering Research Center of Internet of Things for Respiratory Medicine, Shanghai, China; 4 Shanghai Key Laboratory of Lung Inflammation and Injury, Shanghai, China; 5 Shanghai Respiratory Research Institute, Shanghai, China; 6 National Clinical Research Center for Aging and Medicine, Huashan Hospital, Fudan University, Shanghai, China; 7 Department of Pulmonary Medicine, Zhongshan Hospital, Qingpu Branch, Fudan University, Shanghai, China; 8 Jinshan Hospital of Fudan University, Shanghai, China

**Keywords:** Lung cancer, Pulmonary embolism, Rivaroxaban, Low-molecular-weight heparin, Initial anticoagulation

## Abstract

**Background:**

Data that guide selection of differing anticoagulant regimens for specific cancer-associated venous thromboembolism (VTE) are lacking. We aimed to compare the efficacy and safety of rivaroxaban and low-molecular-weight heparin (LMWH) against nonhigh-risk pulmonary embolism (PE) in Chinese lung cancer patients.

**Methods:**

Four hundred forty-six Chinese lung cancer patients with nonhigh-risk PE who initiated treatment with rivaroxaban or LMWH were identified from Zhongshan Hospital database from 2016 to 2020. The primary outcomes were the composite event of VTE recurrence or major bleeding, and all-cause mortality. The secondary outcomes were VTE recurrence, major bleeding and clinically relevant non-major bleeding (CRNMB). Propensity score matching was used to balance baseline covariates. We conducted sensitivity analysis by stabilized inverse probability of treatment weighting and competing risk analysis by a Fine and Gray subdistribution hazard model.

**Results:**

In propensity score-matched cohorts, rivaroxaban was similar to LMWH in the risks of the composite outcome (hazard ratio (HR), 0.73; 95% confidence interval (CI), 0.45–1.21; *P* = 0.22), VTE recurrence (HR, 0.69; 95% CI, 0.36–1.34; *P* = 0.28), major bleeding (HR, 0.79; 95% CI, 0.37–1.68; *P* = 0.54) and CRNMB (HR, 1.13; 95% CI, 0.62–2.09; *P* = 0.69). All-cause mortality was significantly lower in rivaroxaban group than LMWH group (HR, 0.52; 95% CI, 0.36–0.75; *P* < 0.001). The primary and secondary outcomes favored rivaroxaban over LMWH in all the subgroups expect for central PE and intermediate-risk PE. The sensitivity analysis yielded similar results, and competing risk analysis was in accordance with the primary findings.

**Conclusions:**

Rivaroxaban might be a promising alternative to LMWH as initial treatment for nonhigh-risk PE in lung cancer patients.

**Supplementary Information:**

The online version contains supplementary material available at 10.1186/s12959-023-00453-y.

## Introduction

Venous thromboembolism (VTE), including pulmonary embolism (PE) and deep vein thrombosis (DVT), was globally the third most frequent acute cardiovascular syndrome and the second leading cause of mortality in individuals with cancers [1]. The incidence of PE in lung cancer patients was up to 21.5% within twelve months before diagnosis which was 6 times higher than noncancer populations, and lung cancer was an independent predictor of VTE recurrence (recurrence rate: up to 20% within the initial twelve months) [2, 3]. Evidences of PE as the cause of death were found in 10% of lung cancer patients during postmortem examination [4]. Lung cancer patients with PE had significantly lower five-year survival rate, more distant metastases and lower disease progression-free survival than patients without PE [1]. The mortality rate in cancer patients with VTE at six months was 94%, with three times higher risk compared to noncancer patients with VTE (29%)[5]. The higher mortality rate indicated that thrombosis was a marker of more aggressive malignancies or shorter overall survival [6]. With the development of detection and oncotherapy, novel risk factors were found to affect the development of PE, such as positive PD-L1, EML4-ALK or ROS1 rearrangements, abnormal multi-tumor markers, palliative chemo-, radio- and immunotherapy [4, 7, 8]

Low-molecular-weight heparin (LMWH) was the standard treatment for active cancer patients during the acute phase (3–6 months) and secondary prevention of VTE to date [9–11]. Although LMWH decreased the VTE recurrence (up to 52%) with no increase in bleeding compared with warfarin, patients were inclined to switch to oral anticoagulants during the acute phase due to its side effects including injection site pain, local hematomas or allergic reactions [12, 13]. Compared to LMWH, direct oral anticoagulants (DOACs), including dabigatran (thrombin inhibitors), apixaban, edoxaban and rivaroxaban (factor Xa inhibitors), could be given orally at fixed doses and achieve noninferior anticoagulant effect without routine monitoring [9]. It was reported that factor Xa inhibitors were alternative treatment of LMWH to lower the recurrence rate of VTE [14–18]. DOACs were recommended for initial (first week), short-term (3–6 months) and long-term anticoagulation (> 6 months) in active cancer patients with VTE in the guidelines of American Society of Hematology and American Society of Clinical Oncology [10, 19, 20]. Therefore, it is critical to use DOACs that optimize efficacy while minimizing bleeding risk in treating cancer-associated VTE.

Several issues needed to be explored in anticoagulation treatment for cancer-associated VTE. Patients who had concomitant cancer (only 3–9%) or had metastatic cancer receiving chemotherapy (only 15–30%) in phase III clinical trials were not representative for thrombogenesis in cancer patients [21, 22]. Furthermore, some randomized controlled trials on the extended anticoagulation of DOACs excluded patients with highly prothrombotic cancer or high bleeding risks, e.g., gastrointestinal tumors, low platelet count and known major thrombophilia [15, 18, 23]. Finally, limited trails or retrospective studies were designed on one specific cancer with PE and focused on those potential risks of VTE recurrence and major bleeding, e.g., cancer stage, histopathological subtypes, risk stratification of PE, location of PE, platelet count and concomitant DVT, which were worth investigating. Therefore, the aim of our study was to assess the anticoagulant efficacy and safety between rivaroxaban and LMWH for acute nonhigh-risk PE with or without DVT among Chinese lung cancer patients.

## Materials and methods

### Data source

This retrospective cohort study was conducted by using the database of Zhongshan Hospital, Fudan University, Shanghai, China. The hospital is a 2430-bed tertiary hospital treating 20 million people in Shanghai, and its influence radiates throughout China. More than 18,000 inpatients are referred to the Department of Pulmonary and Critical Care Medicine annually. The data elements were available in the electronic medical recording system, such as demographic characteristics, inpatient and outpatient claims, emergency department visits, enrollment history, discharge summaries, medical records, laboratory results, image reports, prescription drug claims and health expenditures for patients by permission. The International Classification of Diseases, Ninth Revision, Clinical Modification (ICD-9-CM) and Tenth Revision, Clinical Modification (ICD-10-CM) record the disease diagnosis and procedures [24, 25]. Patient data which collected from Jan 1, 2016, to Dec 31, 2020 were anonymous, and a unique identification number corresponded with one specific patient to protect personal privacy. All subjects signed informed consent forms, and the study protocol was approved by the Institutional Review Board, Human Subjects Research Protection Program Office (Approval Number: B2021-506R) at Zhongshan Hospital, Fudan University.

### Study population

Patients were included if they were aged 18 years or older and had a diagnosis of active lung cancer and acute symptomatic or incidental PE with or without DVT after objectively confirmation. The index date for both groups was the treatment initiation date of LMWH (mostly nadroparin and enoxaparin) or rivaroxaban. Active lung cancer was defined as a diagnosis of lung cancer within six months before index date, recurrent or metastatic lung cancer, patients treated with systemic therapy or patients with consecutive oncology outpatient visits more than twice within one year [14, 17, 18]. The diagnosis of PE with or without DVT met the criteria including inpatients with a primary or secondary discharge diagnosis per ICD-9 or ICD-10 codes, and outpatients with PE diagnosis and subsequent use of rivaroxaban or LMWH. All study patients were confirmed by computer tomography pulmonary angiography (CTPA) or pulmonary ventilation-perfusion scan for those who were diagnosed as chronic kidney disease or allergic to the contrast agent within 30 days before the first cancer diagnosis or at any time after the first cancer diagnosis. Lower extremity veins ultrasound was detected for DVT. Patients with high-risk PE and hemodynamic instability were excluded, as well as patients with thrombectomy, vena cava filter placement or thrombolysis for acute episode, with a therapeutic dose of any anticoagulants during the 12-month pre-index period, and with anticoagulation therapy less than seven days. Patients with other active cancer or previous chronic thromboembolic disease were excluded from our analysis, as these comorbidities influenced the efficacy and safety of anticoagulation.

### Study design

Lung cancer patients were initiated on LMWH or rivaroxaban after their first PE diagnose, and patients receiving rivaroxaban were compared with those receiving LMWH. Dosages were 15 mg twice daily for the first three weeks, followed by 20 mg once daily for rivaroxaban, and 85 IU per kilogram of body weight every 12 h for nadroparin and 100 AXalU per kilogram of body weight every 12 h for enoxaparin. A dose reduction or discontinuation was specified for different levels of renal impairment and platelet count (discontinuation if platelet counts were < 50,000/mm^3^) until recovery by prescribers. Considering the low proportion and short-term administration of LMWH, patients who received LMWH as bridging therapy (less than 14 days) before rivaroxaban were assigned to rivaroxaban group. The observation period for study population was from index date to the occurrence of any end-point outcome (VTE recurrence, major bleeding and clinically relevant non-major bleeding (CRNMB)), death from any cause, switch to another anticoagulant, treatment discontinuation (a gap less than 7 days was allowed), 12 months after the index date, or end of the study period (Dec 31, 2020). Patients underwent VTE imaging investigations if they were suspected to experience recurrence VTE with new or worse VTE-related symptoms (e.g., dyspnea, acute chest pain, swelling and pain of lower extremities), elevated D-dimer level, or suspicious pulmonary infarction according to regular evaluation. Patients without recurrence VTE underwent routine VTE imaging investigations every 3–6 months after the acute PE episode. The data were collected in person for inpatients with systemic oncotherapy and regular evaluation at Zhongshan Hospital. We kept in close contact with patients who did not be evaluated regularly or their family members by WeChat or telephone interviews at six week intervals on our own initiative. To ensure data integrity, we also collected patient’s data elements from the electronic medical recording database.

### Outcomes

The primary outcomes were the composite outcome of VTE recurrence or major bleeding, and all-cause mortality at 12 months. The secondary outcomes were VTE recurrence, major bleeding and CRNMB at 12 months, respectively. PE recurrence was confirmed based on new intraluminal filling defect on CTPA, a cutoff of a vessel (> 2.5 mm in diameter) on pulmonary angiography, or new perfusion defect (at least 75% of a segment) with corresponding normal ventilation on pulmonary ventilation and perfusion scan. DVT recurrence was defined as new non-compressible venous segment or substantial increase (≥ 4 mm) in the diameter of the thrombus during full compression in a previously abnormal segment on ultrasonography [14]. Major bleeding was identified that it was clinically overt bleeding accompanied with a decrease in the hemoglobin level of more than 2.0 g per deciliter over 24 h; the transfusion of two or more units of packed red cells was necessary; or bleeding occurred at a critical site (e.g., intracranial, intraspinal, intraocular, pericardial, retroperitoneal bleeding) or contributed to death [26]. CRNMB was defined as clinically overt bleeding such as wound hematoma, bruising, gastrointestinal bleeding, hemoptysis, hematuria, and epistaxis, that did not meet the criteria for major bleeding but was associated with medical intervention, unscheduled contact with a physician, treatment interruption, or impairment of daily activities [23]. Death was categorized as a result of PE, bleeding, or other established diagnosis.

### Statistical analysis

The data of patients’ demographics and baseline clinical characteristics were processed by descriptive analyses between LMWH and rivaroxaban groups. We considered patient-, tumor-, and VTE-related potential variables including age, sex, histopathology, cancer TNM stage, type of index PE, risk stratification of PE, concurrent DVT, baseline platelet and creatinine clearance level, time from first cancer diagnosis to index PE, active anticancer treatments, Charlson comorbidity index (CCI) score and medications. Propensity score matching (PSM) was used to estimate the probability of patients receiving rivaroxaban compared to LMWH given a set of measured variables by using multivariable logistic regression model. The matching was 1:1 between rivaroxaban and LMWH groups by a greedy nearest neighbor matching algorithm, with a caliper width equal to 0.2 of the standard deviation of the logit of the propensity score. The balance of baseline variables in two groups was assessed with standardized mean differences (SMD) before and after matching where differences less than 0.1 were considered as well balanced [27].

We compared the incidence rates of primary and secondary outcomes in two groups by using Cox proportional hazards models. An analysis considering death as a competing risk was further performed to compare the risks of time-to-event outcomes in the matched cohorts by obtaining hazard ratios (HRs) and associated 95% confidence intervals (CIs) by a Fine and Gray proportional subdistribution hazard model. Kaplan–Meier cumulative risk curves for time from the index date to first event occurrence were generated to display the distribution of events over time, in which patients were censored if an unexpected end point event happened first.

We assessed heterogeneity of treatment effects with tests of interaction and subgroup analyses which explored the effect of age (≥ 75 or < 75 years), histopathology (adenocarcinoma, squamous cell carcinoma or small cell lung cancer), cancer stage (early/locally advanced or metastatic), type of index PE (peripheral or central PE, single or multiple PE, unilateral or bilateral PE), risk stratification of PE (low or intermediate risk), concurrent DVT (distal DVT, proximal DVT or none), platelet count (≥ 100 000 or < 100 000 per/μl), and incidental or symptomatic PE in the matched population. In sensitivity analyses, stabilized inverse probability of treatment weighting (IPTW) was conducted based on propensity scores for each comparison to adjust for differences in baseline characteristics [28]. Stabilized IPTW was performed in generalized boosted models on the basis of 10,000 regression trees within the TWANG package in R statistical software. Kaplan–Meier curves for the composite outcome were compared in the unmatched, propensity score-matched and IPTW‐weighted populations. Statistical analysis was performed with R version 4.2.0 (R Foundation for Statistical Computing, Vienna, Austria). All reported *P* values were two-sided, and a *P* value < 0.05 was considered a significant difference.

## Results

### Patient characteristics

There were 525 consecutive lung cancer patients who were diagnosed as acute PE and treated with LMWH or rivaroxaban at Zhongshan Hospital, Fudan University from 2016 to 2020. We identified 446 patients with acute low-intermediate risk PE with or without DVT who met our inclusion criteria, of whom 216 (48.4%) received rivaroxaban and 230 (51.6%) received LMWH as anticoagulation treatment within two weeks of their diagnosis (Supplementary Fig. 1). The median duration of usage of anticoagulants was 171 days (interquartile range: 107, 270) in the rivaroxaban group and 152 days (interquartile range: 98, 245) in the LMWH group. There were 31 (13.5%) and 24 (11.1%) patients with previous VTE history, 18 (7.8%) and 13 (6.0%) patients with previous bleeding history, and 8 (3.5%) and 5 (2.3%) patients with thrombophilia in LMWH group and rivaroxaban group, respectively. Patients who were lost to follow-up (11 [2.5%] patients) in overall population were censored in our study. 191 patients treated with rivaroxaban were matched to 191 patients receiving LMWH after 1:1 PSM. Baseline patient characteristics matched by propensity score achieved adequate balance for all the covariates, indicating only small differences between the rivaroxaban and LMWH groups (Table 1). Almost 80% of patients were diagnosed as adenocarcinoma and 88% were diagnosed as stage III or IV. Approximately 50% of them were receiving active oncotherapy, and these features were proved to be risk factors for developing VTE [29]. More than 50% of PE were bilateral and more than 80% of PE involved multiple sites of pulmonary artery after imaging confirmation. Compared to patients receiving LMWH, those receiving rivaroxaban were significantly less likely to be diagnosed with central PE (49 [22.7%] vs 70 [30.4%]; SMD, -0.19), low-intermediate risk PE (35 [16.2%] vs 50 [21.7%]; SMD, -0.15), CCI score ≥ 3 (184 [85.2%] vs 204 [88.7%]; SMD, -0.13), and be currently receiving targeted therapy (42 [19.4%] vs 55 [23.9%]; SMD, -0.11). What’s more, rivaroxaban group had more patients with stage I (22 [10.2%] vs 14 [6.1%]; SMD, 0.14) and low-risk PE (171 [79.2%] vs 169 [73.5%]; SMD, 0.14). There were no significant differences among age, sex, histopathology, level of platelet count (> 100,000/μl) and creatinine clearance (> 60 ml/min), and medications.

**Table 1:** The baseline demographics and clinical characteristics between rivaroxaban and LMWH in the dataset by propensity score matching

Characteristics	Before propensity score matching^a^			After propensity score matching^a^		
	**Rivaroxaban,** **No. (%)** **(*N* = 216)**	**LMWH,** **No. (%)** **(*N* = 230)**	**Standardized mean difference**^**b**^	**Rivaroxaban,** **No. (%)** **(*N* = 191)**	**LMWH,** **No. (%)** **(*N* = 191)**	**Standardized mean difference**^**b**^
**Demographics**						
Mean age (25%,75%), years^c^	65 (61,71)	65 (59, 70)	/	65 (61, 72)	64 (59, 70)	/
Age ≥ 75y	27 (12.5)	35 (15.2)	-0.0822	26 (13.6)	24 (12.6)	0.0317
Gender, female	91 (42.1)	101 (43.9)	-0.0361	85 (44.5)	85 (44.5)	0.0000
**Histopathology of cancer**						
Adenocarcinoma	170 (78.7)	185 (80.4)	-0.0423	154 (80.6)	149 (78.0)	0.0639
Squamous cell carcinoma	28 (13.0)	29 (12.6)	0.0105	23 (12.0)	28 (14.7)	-0.0779
Small cell lung cancer	6 (2.8)	4 (1.8)	0.0632	4 (2.1)	2 (1.0)	0.0637
Others	12 (5.5)	12 (5.2)	0.0148	10 (5.3)	12 (6.3)	-0.0457
**Cancer stage**						
I	22 (10.2)	14 (6.1)	0.1355	17 (8.9)	12 (6.3)	0.0866
II	8 (3.7)	10 (4.3)	-0.0341	8 (4.2)	10 (5.2)	-0.0554
III	28 (13.0)	31 (13.5)	-0.0153	23 (12.0)	26 (13.6)	-0.0468
IV	158 (73.1)	175 (76.1)	-0.0663	143 (74.9)	143 (74.9)	0.0000
**Type of index PE**^**d**^						
Bilateral PE	117 (54.2)	118 (51.3)	0.0574	100 (52.4)	95 (49.7)	0.0525
Central PE	49 (22.7)	70 (30.4)	-0.1850	46 (24.1)	49 (25.7)	-0.0375
Multiple sites of PE	188 (87.0)	191 (83.0)	0.1189	164 (85.9)	159 (83.3)	0.0779
**Risk stratification of PE**^**d**^						
Low	171 (79.2)	169 (73.5)	0.1401	148 (77.5)	142 (74.4)	0.0774
Low to intermediate	35 (16.2)	50 (21.7)	-0.1502	33 (17.3)	40 (20.9)	-0.0995
Intermediate to high	10 (4.6)	11 (4.8)	-0.0073	10 (5.2)	9 (4.7)	0.0249
**Type of index DVT**^**d**^						
Distal DVT	60 (27.8)	49 (21.3)	0.1445	50 (26.2)	46 (24.1)	0.0468
Proximal DVT	26 (12.0)	28 (12.2)	-0.0042	21 (11.0)	26 (13.6)	-0.0805
None	130 (60.2)	153 (66.5)	-0.1294	120 (62.8)	119 (62.3)	0.0107
**Platelet count, per μl**						
> 100,000	208 (96.3)	222 (96.5)	-0.0119	187 (97.9)	186 (97.4)	0.0277
50,000–100,000	8 (3.7)	8 (3.5)	0.0119	4 (2.1)	5 (2.6)	-0.0277
< 50,000	0	0	/	0	0	/
**Creatinine clearance, ml/min**						
> 60	196 (90.8)	210 (91.3)	-0.0194	172 (90.1)	172 (90.1)	0.0000
≤ 60	20 (10.2)	20 (8.7)	0.0194	19 (9.9)	19 (9.9)	0.0000
**Time from first cancer diagnosis to index PE, year**^**e**^						
< 0.5	138 (63.9)	128 (55.7)	0.1715	121 (63.4)	118 (61.8)	0.0327
0.5–1	15 (6.9)	21 (9.1)	-0.0860	13 (6.8)	18 (9.4)	-0.1000
> 1	63 (29.2)	81 (35.2)	-0.1331	57 (29.8)	55 (28.8)	0.0230
**Active anticancer treatment**^**f**^						
Chemotherapy	65 (30.1)	64 (27.8)	0.0494	53 (27.7)	51 (26.7)	0.0228
Targeted therapy	42 (19.4)	55 (23.9)	-0.1129	38 (19.9)	44 (23.1)	-0.0794
Immunotherapy	8 (3.7)	6 (2.6)	0.0580	8 (4.2)	5 (2.6)	0.0832
Others	101 (46.8)	105 (45.7)	0.0222	92 (48.2)	91 (47.6)	0.0105
**Charlson comorbidity index score**						
0	0	0	/	0	0	/
1–2	32 (14.8)	26 (11.3)	0.0988	27 (14.1)	24 (12.0)	0.0590
≥ 3	184 (85.2)	204 (88.7)	-0.1294	164 (85.9)	166 (88.0)	-0.0442
**Medications**						
Antiplatelet use^g^	15 (6.9)	14 (6.1)	0.0337	12 (6.3)	13 (6.8)	-0.0206
NSAIDs	29 (13.4)	35 (15.2)	-0.0525	27 (14.1)	24 (12.6)	0.0461
Corticosteroids	21 (9.7)	17 (7.4)	0.0787	17 (8.9)	15 (7.9)	0.0353

*Abbreviations*: *LMWH* low-molecular-weight heparin, *PE* pulmonary embolism, *DVT* deep vein thrombosis, *NSAIDs* non-steroidal anti-inflammatory drugs

^a^The propensity scores were calculated by all covariates listed in the table

^b^Standardized mean differences of 0.1 or less were considered as well balanced

^c^Propensity score model did not include continuous variables in lieu of the categorized version

^d^Evaluated at the index date

^e^Patients might suffer from subsequent relapse or new metastatic cancer

^f^Included oncotherapy within the 30‐day period preceding the index date

^g^Antiplatelet therapy: aspirin, nonsteroidal anti-inflammatory drugs, or P2Y12 inhibitors

### Primary outcomes

**Fig. 1:**
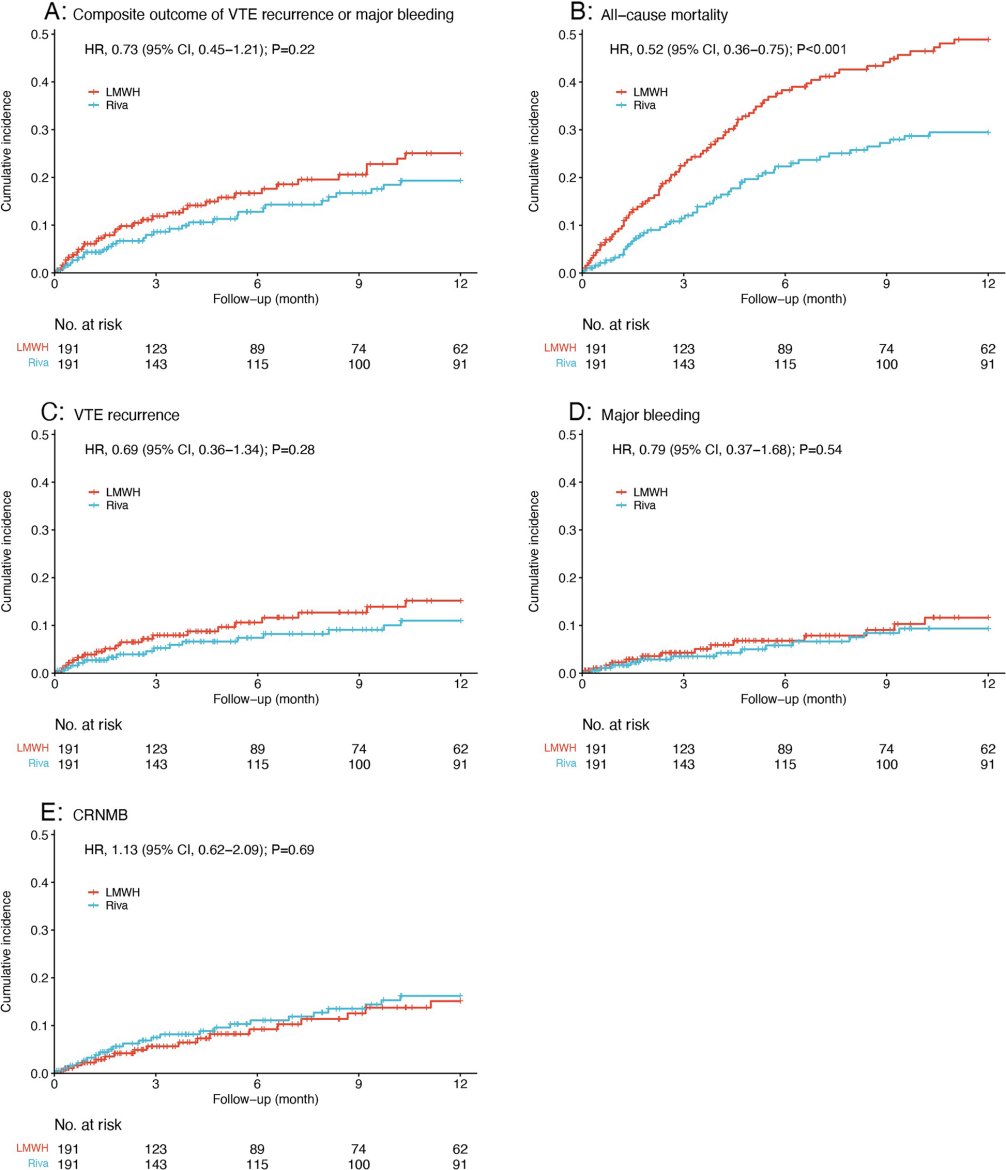
Cumulative incidence of (**A**) composite outcome of VTE recurrence or major bleeding, **B** all-cause mortality, **C** VTE recurrence, **D** major bleeding, and (**E**) CRNMB with rivaroxaban versus LMWH by propensity score-matched analysis. Abbreviations: LMWH, low-molecular-weight heparin; Riva, rivaroxaban; HR, hazard ratio; CI: confidence interval; VTE, venous thromboembolism; CRNMB, clinically relevant non-major bleeding

216 (48.4%) patients reached the primary outcomes in the present study, in whom 140 (31.4%) patients died at 12 months and 76 (17.0%) patients reached the primary composite outcome of VTE recurrence or major bleeding. Patients died from lung cancer (90.7%), PE (2.1%), bleeding (1.4%), and other established or unknown causes (5.7%). Among them, two patients receiving LMWH died from hemoptysis and intracerebral bleeding. The mean survival time was 8.88 [8.41–9.34] months, and death occurred earlier in LMWH group than rivaroxaban group (8.06 [7.36–8.75] months vs 9.68 [9.09–10.27] months, *P* < 0.001) (shown in Supplementary Table 1). The efficacy and safety outcomes were listed in Table 2. The cumulative incidences of the time-to-event outcomes in propensity score-matched population were shown in Fig. 1. Although patients receiving LMWH had higher risk for the composite outcome of VTE recurrence or major bleeding compared to rivaroxaban group, the differences were not significant in ummatched population (33 [15.3%] vs 43 [18.7%]; HR, 0.71; 95% CI, 0.45–1.12; *P* = 0.14) and in PSM population (HR, 0.73; 95% CI, 0.45–1.21; *P* = 0.22). Rivaroxaban group was 46% less likely to have an event of 12-month all-cause mortality compared to LMWH group in unmatched population (52 [24.1%] vs 88 [38.3%]; HR, 0.54; 95% CI, 0.39–0.77; *P* < 0.001), with similar result after PSM analysis (47 [24.6%] vs 77 [40.3%]; HR, 0.52; 95% CI, 0.36–0.75; *P* < 0.001).

**Table 2:** The efficacy and safety outcomes between rivaroxaban and LMWH by unmatched-, PSM-, and competing risk analysis at 12 months

Outcomes^a^	Unmatched population				Propensity score-matched population				Competing risk^b^	
	**Rivaroxaban No. (%)** **(*N* = 216)**	**LMWH** **No. (%)** **(*N* = 230)**	**HR** **(95% CI)**	***P* value**	**Rivaroxaban** **No. (%)** **(*N* = 191)**	**LMWH** **No. (%)** **(*N* = 191)**	**HR** **(95% CI)**	***P* value**	**SHR** **(95% CI)**	***P* value**
**Primary outcomes**^**a**^										
Composite outcome	33 (15.3)	43 (18.7)	0.71 (0.45–1.12)	0.14	29 (15.2)	34 (17.8)	0.73 (0.45–1.21)	0.22	0.83 (0.51–1.37)	0.47
All-cause mortality	52 (24.1)	88 (38.3)	0.54 (0.39–0.77)	< 0.001	47 (24.6)	77 (40.3)	0.52 (0.36–0.75)	< 0.001	0.54 (0.38–0.78)	< 0.001
**Secondary outcomes**^**a**^										
VTE recurrence	18 (8.3)	25 (10.9)	0.67 (0.37–1.23)	0.20	16 (8.4)	20 (10.5)	0.69 (0.36–1.34)	0.28	0.79 (0.41–1.52)	0.47
PE	10 (4.6)	14 (6.1)	0.65 (0.29–1.47)	0.30	9 (4.7)	11 (5.8)	0.70 (0.29–1.69)	0.42	0.81 (0.34–1.95)	0.64
DVT	8 (3.7)	11 (4.8)	0.69 (0.28–1.73)	0.43	7 (3.7)	9 (4.7)	0.69 (0.26–1.86)	0.46	0.77 (0.29–2.06)	0.60
Major bleeding	15 (6.9)	18 (7.8)	0.77 (0.39–1.53)	0.45	13 (6.8)	14 (7.3)	0.79 (0.37–1.68)	0.54	0.92 (0.44–1.96)	0.84
CRNMB	28 (13.0)	22 (9.6)	1.17 (0.67–2.04)	0.59	24 (12.6)	18 (9.4)	1.13 (0.62–2.09)	0.69	1.36 (0.74–2.5)	0.33

*Abbreviations*: *LMWH* low-molecular-weight heparin, *PSM* propensity score matching, *VTE* venous thromboembolism, *PE* pulmonary embolism, *DVT* deep vein thrombosis, *CRNMB* clinically relevant non-major bleeding, *SHR* subdistribution hazard ratio, *CI* Confidence interval

^a^The first event after the index date was identified as the final outcome for patients diagnosed with more than one event

^b^Evaluated by the Fine and Gray proportional subdistribution hazard model considering death as a competing risk in the matched cohorts

### Secondary outcomes

Compared to LMWH, rivaroxaban group was associated with reduced incidence rates of VTE recurrence (8.4% vs 10.5%; HR, 0.69; 95% CI, 0.36–1.34; *P* = 0.28) and major bleeding (6.8% vs 7.3%; HR, 0.79; 95% CI, 0.37–1.68; *P* = 0.54), but there were no significant differences. The risks of PE recurrence (HR, 0.70; 95% CI, 0.29–1.69; *P* = 0.42) and DVT recurrence (HR, 0.69; 95% CI, 0.26–1.86; *P* = 0.46) were similar between rivaroxaban and LMWH groups. The safety outcome of CRNMB favored LMWH over rivaroxaban without significant difference (rivaroxaban vs LMWH: 13.0% vs 9.6%; HR, 1.17; 95% CI: 0.67‐2.04; *P* = 0.59), which was also consistent to the result after matching (rivaroxaban vs LMWH: 12.6% vs 9.4%; HR, 1.13; 95% CI: 0.62‐2.09; *P* = 0.69) (Table 2). Moreover, these results after accounting for competing risk of death were consistent with PSM analysis (rivaroxaban vs LMWH: composite outcome: HR, 0.83; 95% CI, 0.51–1.37; *P* = 0.47; VTE recurrence: HR, 0.79; 95% CI, 0.41–1.52; *P* = 0.47; major bleeding: HR, 0.92; 95% CI, 0.44–1.96; *P* = 0.84), also indicating significant difference in all-cause mortality (HR, 0.54; 95% CI, 0.38–0.78; *P* < 0.001).

### Sensitivity analysis

**Fig. 2:**
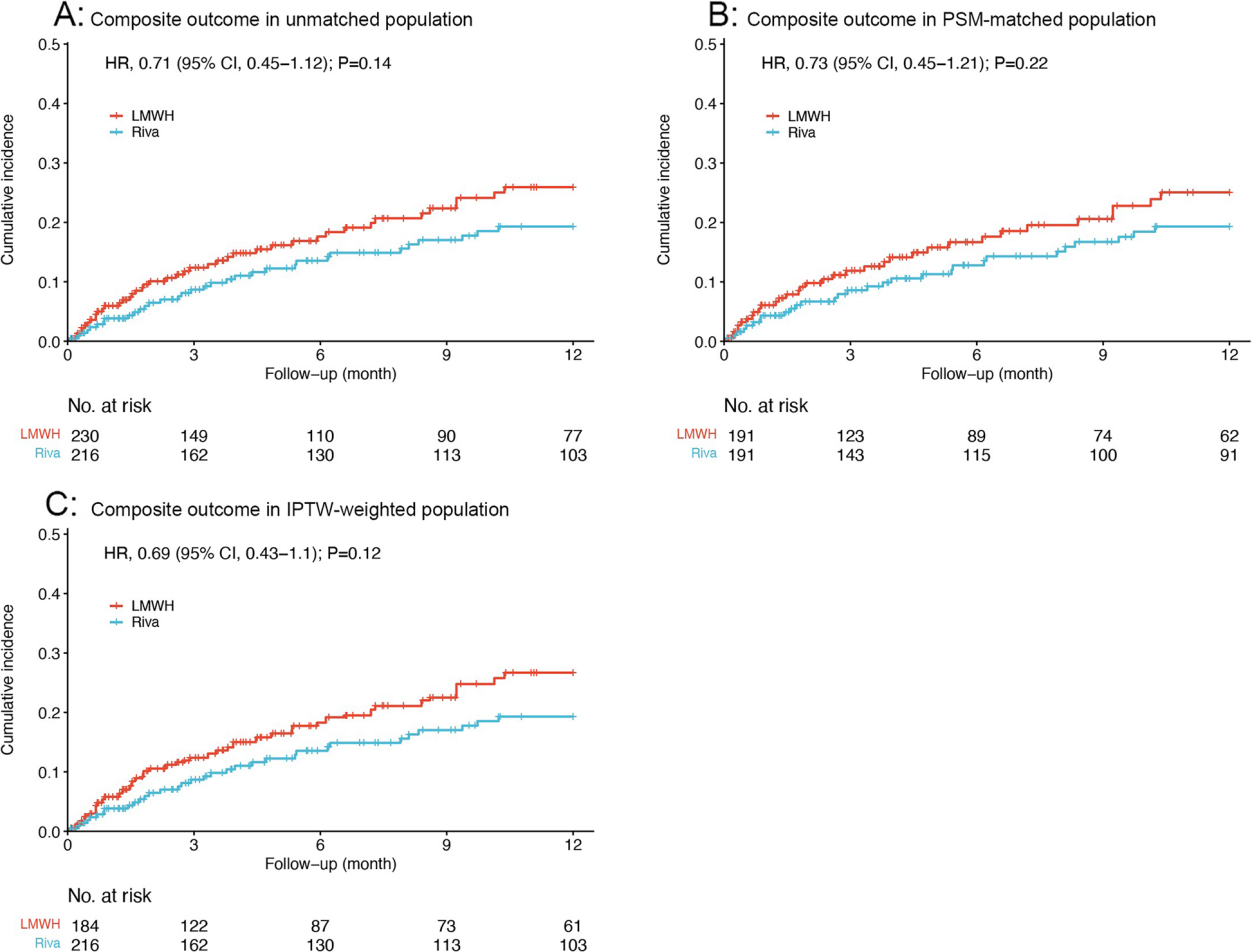
Comparison of composite outcome of VTE recurrence or major bleeding in (**A**) the unmatched analysis, **B** the propensity score-matched analysis, and (**C**) the IPTW–weighted analysis. Abbreviations: VTE, venous thromboembolism; IPTW, inverse probability of treatment weighting; LMWH, low-molecular-weight heparin; Riva, rivaroxaban; HR, hazard ratio; CI: confidence interval

For the sensitivity analysis, the IPTW-weighted population was identified in terms of patient demographics and baseline clinical characteristics which were the same as PSM analysis (Supplementary Table 2). Sensitivity analysis using stabilized IPTW provided results that these outcomes/HRs between rivaroxaban and LMWH groups generally corresponded with the unmatched and PSM-matched analysis (Supplementary Table 3). Although there were no significant risk differences for the composite outcome, VTE recurrence, or major bleeding between rivaroxaban and LMWH groups (HR: 0.69, 95% CI: 0.43‐1.10; HR, 0.61; 95% CI, 0.33–1.14; and HR, 0.82; 95% CI, 0.41–1.66; respectively), rivaroxaban group was at significantly lower risk for all-cause mortality versus LMWH group (HR: 0.54, 95% CI: 0.38‐0.77; *P* < 0.001). The incidence of the composite outcome also showed homogeneity at any point-in-time of observation period (Supplementary Table 3) and a similar rising trend within overall follow-up period (Fig. 2) in the unmatched, PSM and IPTW-adjusted analysis. The primary and secondary outcomes were reconfirmed by different statistical analysis.

### Subgroup assessments

**Fig. 3:**
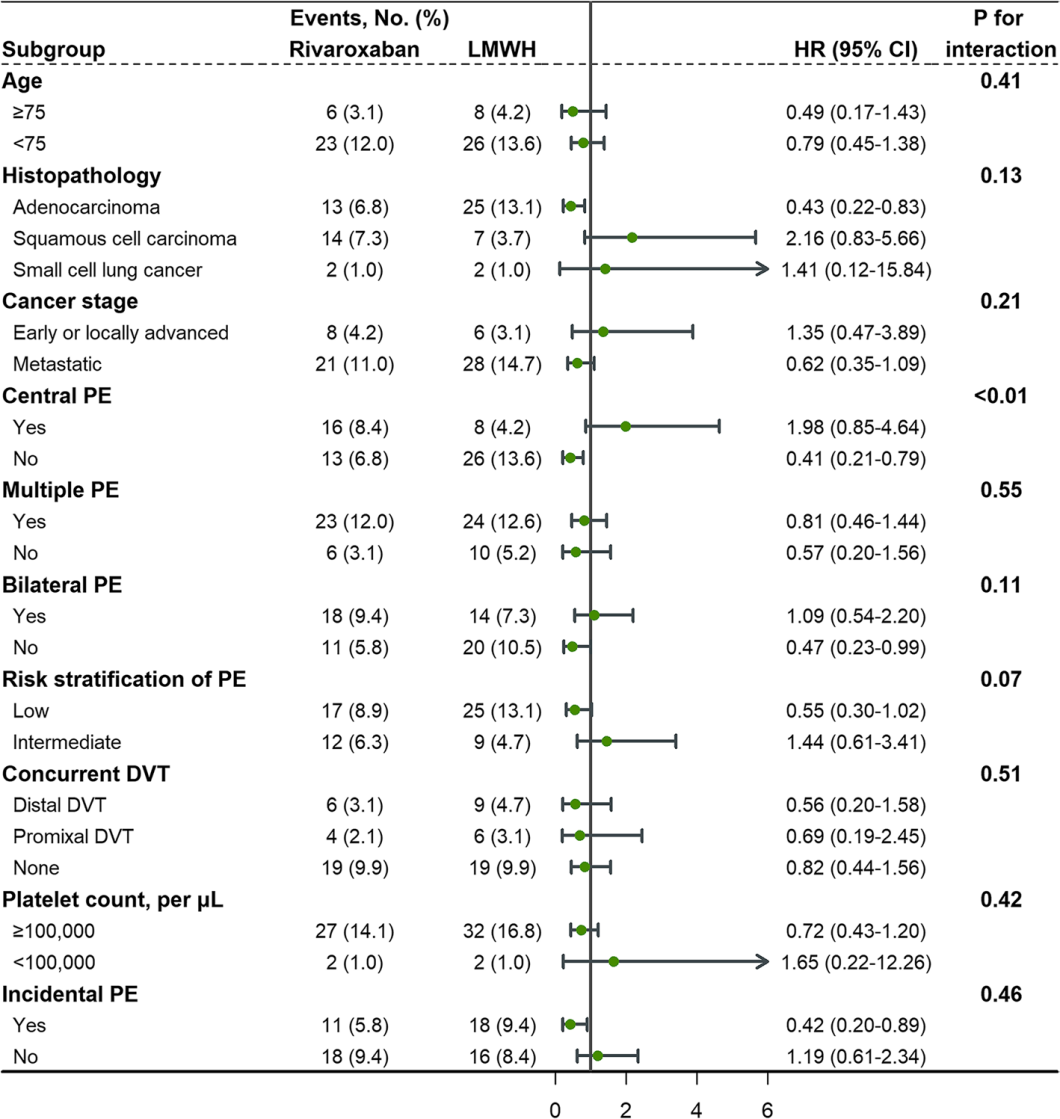
Forest plot depicting hazard ratios of the composite outcome of VTE recurrence or major bleeding between rivaroxaban and LMWH in propensity score-matched population. Abbreviations: PE, pulmonary embolism; DVT, deep vein thrombosis; LMWH, low-molecular-weight heparin; HR, hazard ratio; CI, confidence interval

In the stratified analysis of PE location, patients diagnosed with central PE in rivaroxaban group were at higher risks of the composite outcome (16 patients [8.4%] vs 8 patients [4.2%]; P for interaction = 0.0038) and VTE recurrence (13 patients [6.8%] vs 4 patients [2.1%]; P for interaction < 0.001) than those in LMWH group (Fig. 3 and Supplementary Fig. 2). Intermediate-risk PE patients with rivaroxaban were at 3.7 times higher risk of VTE recurrence than those with LMWH (HR: 3.74, 95% CI: 0.78‐18.02; P for interaction = 0.0125), while the benefit appeared to be more pronounced in the rivaroxaban subgroup with low-risk PE (HR: 0.41, 95% CI: 0.18‐0.91). A similar trend toward an interaction among the risk stratification of PE was found in the incidence of composite outcome (Fig. 3; P for interaction = 0.07). However, there was no significant difference in the safety outcome of major bleeding in all subgroups (Supplementary Fig. 3). Compared to LMWH, rivaroxaban yielded salutary efficacy and safety consistently in all the subgroups examined except for central PE and intermediate-risk PE.

## Discussion

This is the first retrospective cohort study to assess the efficacy and safety of different anticoagulation therapy in Chinese patients with lung cancer-associated VTE by using PSM analysis. Patients treated with rivaroxaban had the decreased risks of composite outcome, VTE recurrence, and major bleeding, and an increased risk of CRNMB without significant differences compared to LMWH users. However, LMWH group had a significantly higher risk of 12-month all-cause mortality compared to rivaroxaban group. The efficacy and safety outcomes after IPTW analysis or competing risk analysis were consistent with primary analysis of PSM-matched population. In subgroup analysis, the primary and secondary outcomes favored rivaroxaban over LMWH in all the subgroups expect for PE location and risk stratification.

For the primary outcomes, rivaroxaban group was 27% less likely to suffer from the composite event of VTE recurrence or major bleeding compared to LMWH group. Unexpectedly, the mortality was significantly higher in LMWH group compared to rivaroxaban group. The trend of all-cause mortality in the present study was similar to an Asian-based retrospective study and two prospective trials, but the other trials with cancer-associated VTE did not show distinct comparisons among vitamin K antagonist, LMWH and DOACs [30–34]. The efficacy outcome of our study was comparable to those previously reported. SELECT-D trials showed less VTE recurrence with rivaroxaban versus dalteparin (4% vs 11%; HR, 0.43; 95% CI, 0.19–0.99), with a similar trend toward a reduced risk in rivaroxaban group of our study (8.4% vs 10.5%; HR, 0.69; 95% CI, 0.36–1.34) [14]. Hokusai and Caravaggio also reported similar results, with a reduction of VTE recurrence in DOACs group (edoxaban vs dalteparin: HR, 0.71; 95% CI, 0.48–1.06; apixaban vs dalteparin: HR, 0.63; 95% CI, 0.37–1.07) [17, 18]. In a 2022 network meta-analysis, rivaroxaban was also associated with noted numerically lower rate of VTE recurrence (OR, 0.41; 95% CI, 0.16–0.95) and higher rate of CRNMB (OR, 4.09; 95% CI, 1.79–10.59) compared with dalteparin, which also accorded with the findings summarized by Overvad et al. [34, 35].

For the safety outcomes, there was no significant difference between DOAC and LMWH in the incidence of major bleeding in our study, which was in accordance with SELECT-D and Caravaggio trials but in contrast to Hokusai trials favoring LMWH (edoxaban vs dalteparin: HR 1.77, 95% CI 1.03–3.04, *P* = 0.04). SELECT-D trials indicated a significantly increased risk of CRNMB in rivaroxaban (HR, 3.76; 95% CI, 1.63–8.69), which corresponded to our study with a rising tendency of CRNMB in rivaroxaban group with no significant difference (HR, 1.13; 95% CI, 0.62–2.09). It was reported that rivaroxaban was ranked the least safe drug in CRNMB compared with dalteparin (OR, 4.09; 95% CI, 1.79–10.59) and other DOACs (rivaroxaban vs apixaban: OR, 2.73; 95% CI, 1.08–7.71; rivaroxaban vs edoxaban: OR, 2.99; 95% CI, 1.21–8.26) [34]. It reflected a special hemorrhagic vulnerability of cancer tissue to rivaroxaban rather than the specific predilection for specific cancer. Our clinical findings were also consistent with the effect estimates derived from a meta-analysis including more than 35,000 patients in observational studies and more than 2,000 patients in clinical trials [36].

Three additional aspects of our results warranted comment. First, these results consistently demonstrated that rivaroxaban had the improved efficacy, noninferior safety of major bleeding and increased risk of CRNMB compared to LMWH. The enhanced antithrombotic effect of rivaroxaban was associated with its high peak serum anti-Xa activity and a greater perturbation of coagulation through high-selectively inhibiting prothrombinase activity and factor Xa without any cofactors, whereas LMWH only inhibited the antithrombin of activated factor X [37]. Rivaroxaban was ranked the least safe in CRNMB due to its local and systemic effect on gastrointestinal bleeding, as well as its high peak-to-trough ratio with a less favorable safety effect [38]. Other potential reasons for more CRNMB included lower mortality, greater length of time in therapeutic range, more complications with high bleeding risks and more drug interactions that altered the serum level and bioavailability of rivaroxaban. CRNMB might increase the propensity for major bleeding and lead to anticoagulation interruptions which increased VTE recurrence. It suggested further research to reduce CRNMB through risk stratification, refinement of the anticoagulant regimens, and minimizing drug interactions by reviewing concomitant medications. Second, the all-cause mortality in rivaroxaban group (24.1%) at 12 months was significantly lower than LMWH group (38.3%) in the present study, and the mortality with rivaroxaban (18.9%) at 6 months in our study was also lower than that with rivaroxaban (25%) in the SELECT-D trial. Our study focused on Chinese lung cancer patients, and the mortality rate was comparable to an Asian-based retrospective study [31]. The lower mortality of rivaroxaban compared with other trials might be due to ethnic differences, specific cancer types and risk stratification of PE between populations. Khorana et al. reported rivaroxaban had a better overall survival than LMWH without decreased VTE recurrence or major bleeding rates [39]. However, LMWH group in our study was more closely associated with high risk factors of VTE, and patients with more aggressive or extensive cancer were inclined to be administrated to LMWH group due to selection bias by prescribers. It was reported that poorer performance status and higher bleeding risks were associated with higher all-cause mortality, and those residual variables were worthy of investigation [40]. Third, the benefit of rivaroxaban in the efficacy and safety was consistent in all the subgroups except for central PE and intermediate-risk PE. In the present study, the composite outcome occurred earlier in central PE group than peripheral PE group (mean time to composite outcome: 9.31 [8.37–10.25] vs 10.63 [10.21–11.04], *P* = 0.006), and central PE patients exhibited higher rate of concurrent DVT (34.8% vs 45.3%, *P* = 0.046). Central PE patients were also reported to be at greater risk for right ventricular dysfunction, recurrent VTE, hemodynamic consequences and worse prognosis [9, 41, 42]. However, there was no significant difference in the overall mortality between central PE and peripheral PE (HR: 0.93, 95% CI: 0.61‐1.42, *P* = 0.724), which could be attributed to common test of D-dimer and anticoagulation in time. On the other hand, hemodynamically stable patients with elevated level of brain natriuretic peptide or right ventricular dysfunction were at increased risk for early recurrent VTE [43–46]. Central PE was also identified to be the strongest individual predictor for right ventricular pressure overload [42]. These findings reflected the close relationship between the localization of the emboli and the risk stratification of PE. Thus, they seemed to interact with each other since central PE was presenting more acute with less time of the right ventricle for sudden pressure overload and adaptation. In summary, physicians should pay more attention to patients with central PE or intermediate-high risk PE who were associated with heavier thrombotic burden, higher incidence of early VTE recurrence and prothrombotic activity of tumor cells, which could lead to increase of adverse events and oncotherapy interruption. Therefore, it was essential to find who might profit from rivaroxaban in terms of race, clinical characteristics of cancer and PE, relevant drug interactions, and concomitant cancer treatment. These results provided additional arguments for the benefits and risks from broader use of DOACs in lung cancer patients with PE.

Since there were no head-to-head comparative trials between rivaroxaban and LMWH in lung cancer with PE, our study was the first retrospective analysis on the anticoagulation therapy selection for Chinese patients with lung cancer-associated PE. To control confounding bias, we performed PSM to balance baseline differences and stabilized IPTW for sensitivity analysis. The outcomes after stabilized IPTW or adjusting for competing risks remained consistent with PSM analysis, proving the stability of these results among the unmatched, PSM-matched and IPTW-weighted population. Due to indefinite anticoagulation for cancer-associated VTE, the follow-up period of our study (12 months) was longer than that of most trials (3–6 months) leaving uncertainty about the anticoagulant effect beyond this period. Furthermore, we did not exclude the patients with high bleeding risks or poor performance status, making results more accordant to routine clinical practice. Treatment with rivaroxaban yielded superiority of the efficacy and safety over LMWH expect for the subgroups of central PE and intermediate-risk PE, which endorsed caution with use of rivaroxaban in special types of PE patients. Our finding also indicated rivaroxaban could be a more convenient alternative to LMWH as the initial treatment without prior 3–6 months use of LMWH. Considering that cancer patients with VTE was heterogenous and complex, our study focused on lung cancer-specific PE in Chinese patients, and important future directions must include cancer-specific anticoagulation selection for the initial treatment and long-term management.

Our study had some limitations. As a single center retrospective cohort study, our findings focused on Chinese lung cancer patients with nonhigh-risk PE receiving rivaroxaban or LMWH with an absence of generalization. Information on prescribed drugs from database was likely to overestimate patients’ compliance, and the anticoagulant effect was directly influenced by the normalization of treatment, patients’ preference and medication selection bias. Furthermore, between-group difference with respect to accumulative time interval of suspension of anticoagulants due to adverse events of oncotherapy may impact these outcomes between the DOAC and LMWH receivers. Indeed, LMWH group had high proportion of central PE, intermediate-risk PE, CCI score ≥ 3, and receiving palliative oncotherapy during the study period, which suggested that the more advanced cancer stage and heavier thrombotic burden might contributed to the increased risks of VTE recurrence and mortality. Death mostly occurred in patients with those clinical characteristics who were inclined to be administered to LMWH group, while patients with better prognosis were more likely to be treated with rivaroxaban, increasing the likelihood of lower mortality rate for the cohort. Finally, clinicians should be more cautious about the result of lower mortality associated with rivaroxaban. The survival difference in our study might be due to selection bias by prescribers, and the true rate of VTE or bleeding related death might be underestimated as a substantial proportion of patients did not die at hospital. Residual confounding variables which were not recorded in our study, such as cancer progression, performance status, tumor response evaluation and gene mutations, might be a limitation and influence the survival outcome of all-cause mortality. The result of lower mortality with rivaroxaban should be verified in prospective randomized clinical trials.

## Conclusion

This is the first cohort study comparing rivaroxaban and LMWH in Chinese lung cancer patients with nonhigh-risk PE in the real-world setting. Rivaroxaban group experienced lower rates of the composite outcome, VTE recurrence and major bleeding but a rising trend of CRNMB compared to LMWH group by using PSM analysis. Nonetheless, rivaroxaban was superior over LMWH at all-cause mortality, which might be driven by heavy thrombotic burden or selection bias in LMWH group. The superiority of efficacy and safety in rivaroxaban group was consistent in all the subgroups except for central PE and intermediate-risk PE. These outcomes were confirmed after accounting for potential variables, competing risk of death and sensitivity analysis with IPTW. Rivaroxaban could be alternative to LMWH for the initial treatment against nonhigh-risk PE. Further prospective investigations are warranted to explore the benefit-risk ratio of DOACs in lung cancer with PE.

## Supplementary Information

**Additional file 1: Supplementary Table 1.** Causes and timing of death in overall population, rivaroxaban group and LMWH group. **Supplementary Table 2.** The baseline demographics and clinical characteristics between rivaroxaban and LMWH by the stabilized IPTW analysis. **Supplementary Table 3.** The primary and secondary outcomes between rivaroxaban and LMWH by unmatched, propensity score-matched and IPTW-weighted analysis at 12 months. **Supplementary Fig. 1.** Patient enrollment and follow-up. **Supplementary Fig. 2.** Forest plot depicting hazard ratios of the efficacy outcome of VTE recurrence between rivaroxaban and LMWH in propensity score-matched population. **Supplementary Fig. 3.** Forest plot depicting hazard ratios of the safety outcome of major bleeding between rivaroxaban and LMWH in propensity score-matched population.

## Data Availability

The datasets analysed during the current study available from the corresponding author on reasonable request.

## Abbreviations

VTE: Venous thromboembolism
PE: Pulmonary embolism
DVT: Deep vein thrombosis
LMWH: Low-molecular-weight heparin
DOACs: Direct oral anticoagulants
ICD-9-CM: The International Classification of Diseases, Ninth Revision, Clinical Modification
ICD-10-CM: The International Classification of Diseases, Tenth Revision, Clinical Modification
CTPA: Computer tomography pulmonary angiography
CRNMB: Clinically relevant non-major bleeding
CCI: Charlson comorbidity index
PSM: Propensity score matching
SMD: Standardized mean differences
IPTW: Inverse probability of treatment weighting
HR: hazard ratio
CI: Confidence interval
