# Supplementary material for: Comparison of the efficacy and safety of rivaroxaban and low-molecular-weight heparin in Chinese lung cancer patients with nonhigh-risk pulmonary embolism
Source: Thromb J. 2023 Feb 2;21:16. doi: 10.1186/s12959-023-00453-y (PMC9893535; doi:10.1186/s12959-023-00453-y)
Supplement: Supplementary file 1 — Additional file 1: Supplementary Table 1. Causes and timing of death in overall population, rivaroxaban group and LMWH group. Supplementary Table 2. The baseline demographics and clinical characteristics between rivaroxaban and LMWH by the stabilized IPTW analysis. Supplementary Table 3. The primary and secondary outcomes between rivaroxaban and LMWH by unmatched, propensity score-matched and IPTW-weighted analysis at 12 months. Supplementary Fig. 1. Patient enrollment and follow-up. Supplementary Fig. 2. Forest plot depicting hazard ratios of the efficacy outcome of VTE recurrence between rivaroxaban and LMWH in propensity score-matched population. Supplementary Fig. 3. Forest plot depicting hazard ratios of the safety outcome of major bleeding between rivaroxaban and LMWH in propensity score-matched population. [file 12959_2023_453_MOESM1_ESM.docx]

**Supplementary material**

Comparison of the efficacy and safety of rivaroxaban and low-molecular-weight heparin in Chinese lung cancer patients with nonhigh-risk pulmonary embolism

Yijun Song^1#^, Dawei Yang^1,2,3#^, Dongni Hou^1^, Jun She^1*^, Yuanlin Song^1,2,3,4,5,6,7,8*^

^1^ Department of Pulmonary and Critical Care Medicine, Zhongshan Hospital, Fudan University, Shanghai, China;

^2^ Department of Pulmonary and Critical Care Medicine, Zhongshan Hospital (Xiamen), Fudan University, Xiamen, China;

^3^ Shanghai Engineering Research Center of Internet of Things for Respiratory Medicine, Shanghai, China;

^4^ Shanghai Key Laboratory of Lung Inflammation and Injury, Shanghai, China;

^5^ Shanghai Respiratory Research Institute, Shanghai, China;

^6^ National Clinical Research Center for Aging and Medicine, Huashan Hospital, Fudan University, Shanghai, China;

^7^ Department of Pulmonary Medicine, Zhongshan Hospital, Qingpu Branch, Fudan University, Shanghai, China;

^8^ Jinshan Hospital of Fudan University, Shanghai, China

^*^ **Corresponding to:** Jun She; Yuanlin Song. Department of Pulmonary and Critical Care Medicine, Zhongshan Hospital, Fudan University, 180 Fenglin Road, Shanghai 200032, China. Email: [shejuncn@aliyun.com](mailto:shejuncn@aliyun.com); [ylsong70@163.com](mailto:ylsong70@163.com).

^#^ YJS and DWY contributed equally.

Supplementary Table 1. Causes and timing of death in overall population, rivaroxaban group and LMWH group

Supplementary Table 2. The baseline demographics and clinical characteristics between rivaroxaban and LMWH by the stabilized IPTW analysis

Supplementary Table 3. The primary and secondary outcomes between rivaroxaban and LMWH by unmatched, propensity score-matched and IPTW-weighted analysis at 12 months

Supplementary Figure 1. Patient enrollment and follow-up

Supplementary Figure 2. Forest plot depicting hazard ratios of the efficacy outcome of VTE recurrence between rivaroxaban and LMWH in propensity score-matched population

Supplementary Figure 3. Forest plot depicting hazard ratios of the safety outcome of major bleeding between rivaroxaban and LMWH in propensity score-matched population

Supplementary Table 1. Causes and timing of death in overall population, rivaroxaban group and LMWH group

| **Cause and timing of death** | **Overall**  **N=446** | **Rivaroxaban**  **N=216** | **LMWH**  **N=230** | **P value** |
| --- | --- | --- | --- | --- |
| **Death from all causes at 12 months, No. (%)** | 140 (31.4) | 52 (24.1) | 88 (38.3) | <0.001 |
| **Cause of death, No. (%)** |  |  |  |  |
| Lung cancer | 127 (90.7) | 47 (90.4) | 80 (90.9) | NA |
| PE-related | 3 (2.1) | 2 (3.8) | 1 (1.1) | NA |
| Bleeding | 2 (1.4) | 0 (0) | 2 (2.3) | NA |
| Others | 8 (5.7) | 3 (5.7) | 5 (5.7) | NA |
| **Mean survival time, months** | 8.88 [8.41-9.34] | 9.68 [9.09-10.27] | 8.06 [7.36-8.75] | <0.001 |

Abbreviations: PE, pulmonary embolism; LMWH, low-molecular-weight heparin.

Most patients died from lung cancer, and other causes of death were categorized as PE, bleeding, other established or unknown causes. The mean survival time to death was 8.88 months, and death occurred earlier in LMWH group than rivaroxaban group.

Supplementary Table 2: The baseline demographics and clinical characteristics between rivaroxaban and LMWH by the stabilized IPTW analysis

| **Characteristics** | **LMWH, %**  **(N=184.2)** | **Rivaroxaban, %**  **(N=216)** | ***P* value** | **Standardized**  **mean difference^a^** |
| --- | --- | --- | --- | --- |
| **Demographics** |  |  |  |  |
| Age ≥ 75y | 12.4 | 12.5 | 0.972 | 0.003 |
| Gender, female | 39.9 | 42.1 | 0.642 | 0.046 |
| **Histopathology of cancer** |  |  |  |  |
| Adenocarcinoma | 78.9 | 78.7 | 0.933 | 0.067 |
| Squamous cell carcinoma | 14.2 | 13.0 |  |  |
| Small cell lung cancer | 2.0 | 2.8 |  |  |
| Others | 4.9 | 5.5 |  |  |
| **Cancer stage** |  |  |  |  |
| I | 8.6 | 10.2 | 0.864 | 0.087 |
| II | 5.0 | 3.7 |  |  |
| III | 12.2 | 13.0 |  |  |
| IV | 74.2 | 73.1 |  |  |
| **Type of index PE^b^** |  |  |  |  |
| Bilateral PE | 52.1 | 54.2 | 0.671 | 0.042 |
| Central PE | 24.0 | 22.7 | 0.743 | 0.031 |
| Multiple sites of PE | 85.6 | 87.0 | 0.662 | 0.043 |
| **Risk stratification of PE^b^** |  |  |  |  |
| Low | 78.5 | 79.2 | 0.963 | 0.026 |
| Low to intermediate | 17.1 | 16.2 |  |  |
| Intermediate to high | 4.4 | 4.6 |  |  |
| **Type of index DVT^b^** |  |  |  |  |
| Distal DVT | 25.2 | 27.8 | 0.835 | 0.060 |
| Proximal DVT | 12.1 | 12.0 |  |  |
| None | 62.7 | 60.2 |  |  |
| **Platelet count, per μl** |  |  |  |  |
| >100,000 | 96.9 | 96.3 | 0.706 | 0.035 |
| 50,000-100,000 | 3.1 | 3.7 |  |  |
| <50,000 | 0 | 0 |  |  |
| **Creatinine clearance, ml/min** |  |  |  |  |
| >60 | 91.4 | 90.7 | 0.821 | 0.022 |
| ≤60 | 8.6 | 9.3 |  |  |
| **Time from first cancer diagnosis to index PE, year^c^** |  |  |  |  |
| <0.5 | 63.0 | 63.9 | 0.732 | 0.076 |
| 0.5-1 | 9.0 | 6.9 |  |  |
| >1 | 28.0 | 29.2 |  |  |
| **Active anticancer treatment^d^** |  |  |  |  |
| Chemotherapy | 30.5 | 30.1 | 0.937 | 0.063 |
| Targeted therapy | 19.4 | 19.4 |  |  |
| Immunotherapy | 2.6 | 3.7 |  |  |
| Others | 47.5 | 46.8 |  |  |
| **Charlson comorbidity index score** |  |  |  |  |
| 0 | 0 | 0 | 0.629 | 0.075 |
| 1-2 | 13.6 | 14.8 |  |  |
| ≥3 | 86.4 | 85.2 |  |  |
| **Medications** |  |  |  |  |
| Antiplatelet use^e^ | 6.1 | 6.9 | 0.736 | 0.033 |
| NSAIDs | 12.5 | 13.4 | 0.784 | 0.027 |
| Corticosteroids | 7.2 | 9.7 | 0.367 | 0.090 |

Abbreviations: LMWH, low-molecular-weight heparin; IPTW, inverse probability of treatment weighting; PE, pulmonary embolism; DVT, deep vein thrombosis; NSAIDs, non-steroidal anti-inflammatory drugs.

^a^Standardized mean differences of 0.1 or less were considered as well balanced.

^b^Evaluated at the index date.

^c^Patients might suffer from subsequent relapse or new metastatic cancer.

^d^Included oncotherapy within the 30‐day period preceding the index date.

^e^Antiplatelet therapy: aspirin, nonsteroidal anti-inflammatory drugs, or P2Y12 inhibitors.

Supplementary Table 3: The primary and secondary outcomes between rivaroxaban and LMWH by unmatched, propensity score-matched and IPTW-weighted analysis at 12 months

| **Outcomes** | **Unmatched (%)** | | **Propensity score-matched (%)** | | **IPTW-weighted (%)** | | | |
| --- | --- | --- | --- | --- | --- | --- | --- | --- |
|  | **Rivaroxaban** | **LMWH** | **Rivaroxaban** | **LMWH** | **Rivaroxaban** | **LMWH** | **HR (95% CI)** | ***P* value** |
| Composite outcome- VTE recurrence or major bleeding |  |  |  |  |  |  |  |  |
| 1 month | 3.7 | 5.7 | 4.2 | 5.8 | 3.7 | 5.5 | 0.65 (0.27-1.60) | 0.36 |
| 3 month | 7.9 | 10.9 | 7.9 | 10.5 | 7.9 | 11.0 | 0.67 (0.36-1.27) | 0.22 |
| 6 month | 11.6 | 14.8 | 11.0 | 13.6 | 11.6 | 15.0 | 0.71 (0.42-1.20) | 0.20 |
| 9 month | 13.9 | 17.4 | 13.6 | 15.7 | 13.9 | 17.3 | 0.72 (0.44-1.17) | 0.18 |
| 12 month | 15.3 | 18.7 | 15.2 | 17.8 | 15.2 | 19.4 | 0.69 (0.43-1.10) | 0.12 |
| All-cause mortality | 24.1 | 38.3 | 24.6 | 40.3 | 24.1 | 38.5 | 0.54 (0.38-0.77) | <0.001 |
| VTE recurrence | 8.3 | 10.9 | 8.4 | 10.5 | 8.3 | 12.0 | 0.61 (0.33-1.14) | 0.12 |
| Major bleeding | 6.9 | 7.8 | 6.8 | 7.3 | 6.9 | 7.4 | 0.82 (0.41-1.66) | 0.58 |
| CRNMB | 13.0 | 9.6 | 12.6 | 9.4 | 13.0 | 8.9 | 1.27 (0.71-2.25) | 0.42 |

Abbreviations: LMWH, low-molecular-weight heparin; IPTW, inverse probability of treatment weight; HR, hazard ratio; CI: confidence interval; VTE, venous thromboembolism; CRNMB, clinically relevant non-major bleeding.

Supplementary Figure 1. Patient enrollment and follow-up


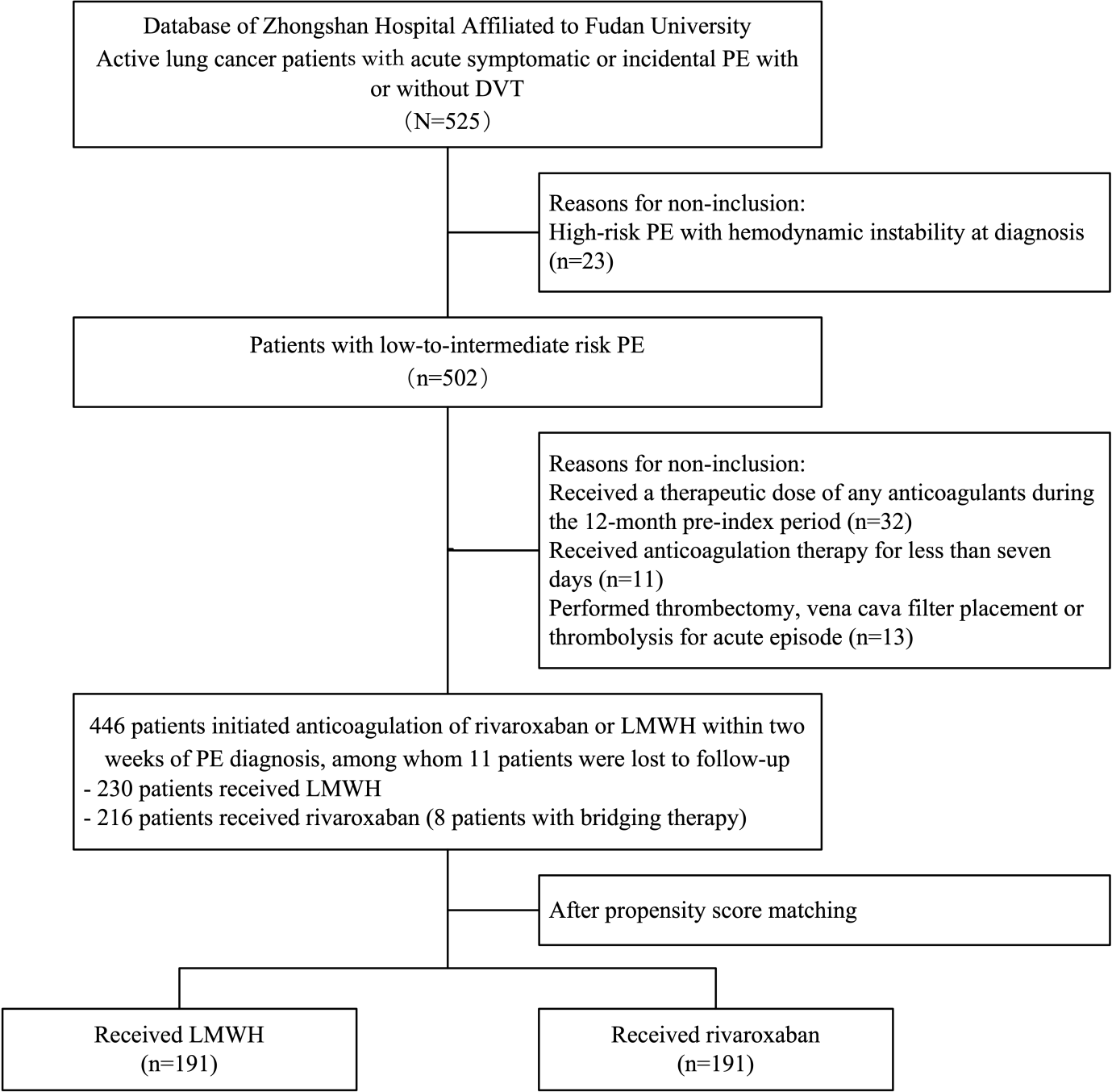


There were 446 patients with acute low-intermediate risk PE who met our inclusion criteria in 525 consecutive lung cancer patients treated with LMWH or rivaroxaban at Zhongshan Hospital from 2016 to 2020. Among them, 216 received rivaroxaban and 230 received LMWH within two weeks of PE diagnosis.

Abbreviations: PE, pulmonary embolism; DVT, deep vein thrombosis; LMWH, low-molecular-weight heparin.

Supplementary Figure 2: Forest plot depicting hazard ratios of the efficacy outcome of VTE recurrence between rivaroxaban and LMWH in propensity score-matched population


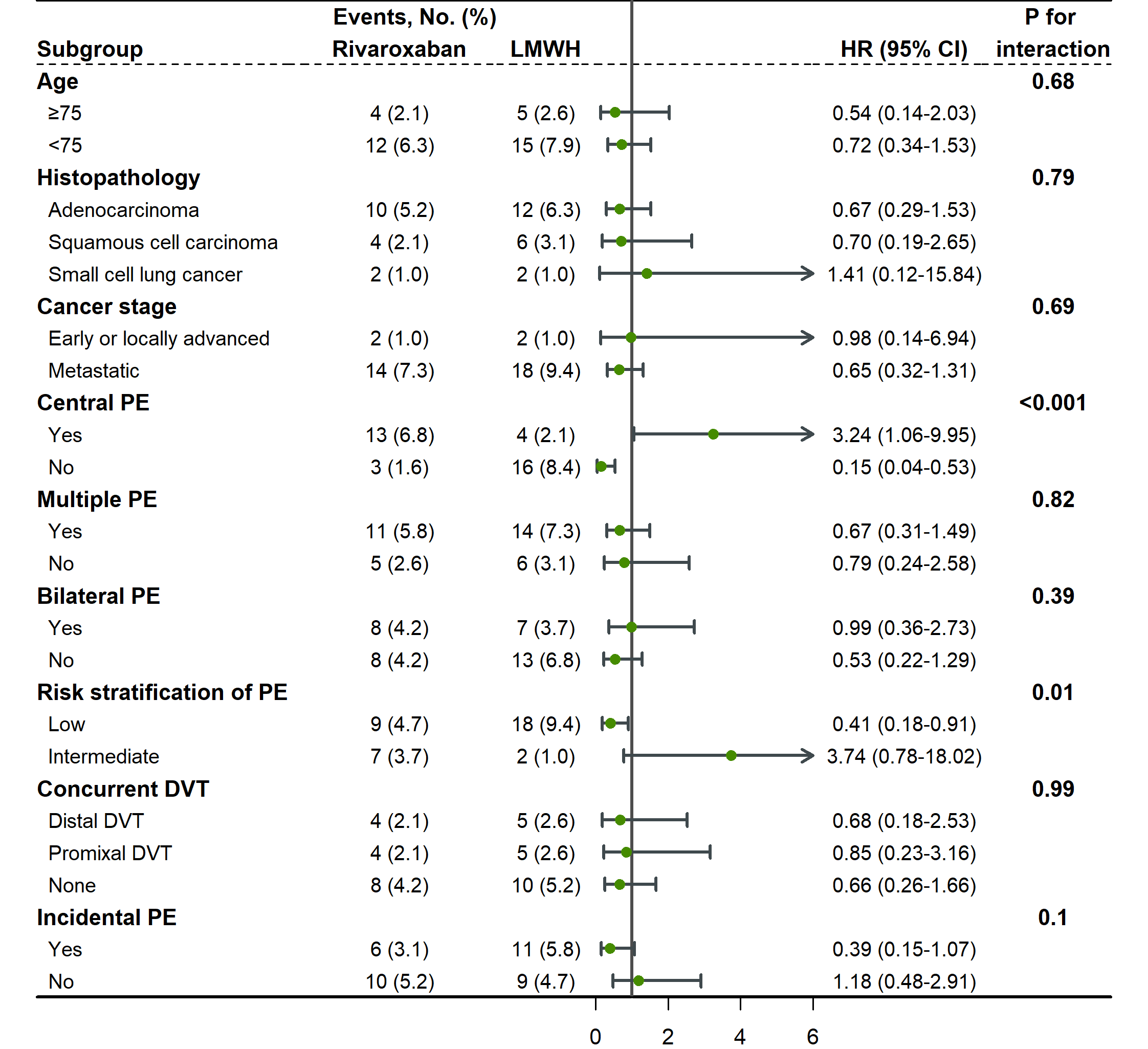


Abbreviations: PE, pulmonary embolism; DVT, deep vein thrombosis; LMWH, low-molecular-weight heparin; HR, hazard ratio; CI, confidence interval.

Supplementary Figure 3: Forest plot depicting hazard ratios of the safety outcome of major bleeding between rivaroxaban and LMWH in propensity score-matched population


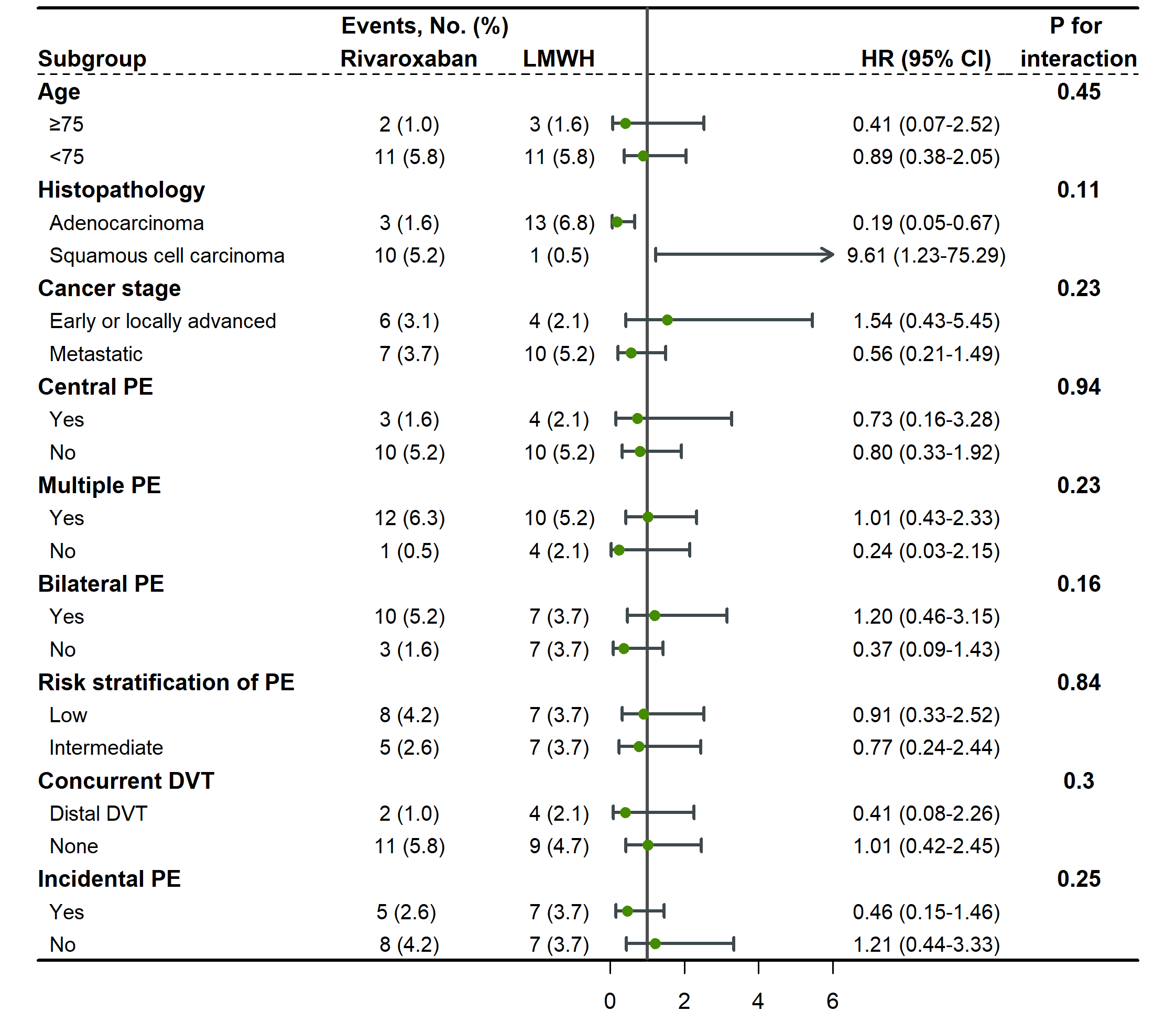
Abbreviations: PE, pulmonary embolism; DVT, deep vein thrombosis; LMWH, low-molecular-weight heparin; HR, hazard ratio; CI, confidence interval.
